# The application value of preoperative magnetic resonance cholangiopancreatography in predicting the success of endoscopic retrograde cholangiopancreatography stone removal

**DOI:** 10.3389/fmed.2026.1759986

**Published:** 2026-03-09

**Authors:** Xuhua Xiao, Xiaoguang Shi, Yan Zhang, Linzhen Li, Zhipeng Zhou

**Affiliations:** 1 Departments of Gastroenterology, The Affiliated Hospital of Guilin Medical University, Guilin, China; 2 Departments of Gastroenterology, First Affiliated Hospital of Wannan Medical College, Wuhu, Anhui Province, China; 3 Department of Radiology, The Affiliated Hospital of Guilin Medical University, Guilin, Guangxi, China

**Keywords:** common bile duct stones, ERCP, MRCP, removal, stone diameter

## Abstract

**Background and purpose:**

Common bile duct stones (CBDS) are a common disease. Endoscopic retrograde cholangiopancreatography (ERCP) is currently recognized as the preferred treatment method for CBDS. However, there are cases that cannot be removed through ERCP. Therefore, the aim of this study is to explore the value of magnetic resonance cholangiopancreatography (MRCP) in assessing the success of CBDS removal through ERCP.

**Patients and methods:**

A total of 432 CBDS patients were include in this study. According to whether the stone removal was successful in ERCP, the patients were divided into the successful stone removal group and the failed stone removal group. The differences in MRCP-related parameters between the two groups were analyzed.

**Results:**

This multi-center study included a total of 175 male CBDS patients and 257 female CBDS patients. A total of 395 CBDS patients successfully removed stones through ERCP, with a success rate of 91.44%. There were significant differences in the stone diameter (*p* < 0.001), the widest diameter of the CBD (*p* < 0.001), and the widest diameter of the CBD/stone diameter (*p* < 0.001) between success group and failure group. But, there was no significant difference in the number of single stone patients (*p* = 0.174). Binary multivariate logistic regression analysis revealed that only stone diameter >15 mm was significantly associated with ERCP stone extraction failure (OR = 11.229, 95% CI: 1.576–80.033, *p* = 0.016). ROC curve analysis demonstrated that this cutoff value had excellent predictive performance, with an area under the curve (AUC) of 0.94, a maximum Youden’s index of 0.749, 100% sensitivity, and 75% specificity.

**Conclusion:**

The results of this study confirm that preoperative MRCP plays a significant role in assessing the success of ERCP stone removal in CBDS patients. The stone diameter >15 mm was identified as an independent risk factor.

## 1 Introduction

Common bile duct stones (CBDS) are a frequent clinical condition. As the stones block the common bile duct (CBD), it leads to impaired bile excretion. Patients may experience abdominal pain, jaundice, and even acute cholangitis (1–3). Since CBDS are more common among the elderly population, with the aging of the global population, the incidence of CBDS is expected to increase in the future. Among the Asian population, primary CBDS are more common, usually caused by biliary tract infection and bile stasis (4). The latest guidelines recommend using endoscopic ultrasonography (EUS) or magnetic resonance cholangiopancreatography (MRCP) for the diagnosis of CBDS. A meta-analysis published in 2015 showed that the sensitivity and specificity for MRCP 93 and 96%, and for EUS were 95 and 97% (5). Although studies have shown that EUS has a higher overall diagnostic advantage than MRCP, and a higher detection rate for small stones than MRCP (6), EUS is not as convenient to operate as MRCP. It is not widely used in many hospitals, while MRCP is almost universally available in most hospitals. Although CT is also a diagnostic method for CBDS, due to the presence of radiation exposure and the lower diagnostic accuracy compared to MRCP and EUS, the current guidelines do not recommend it as the preferred method for diagnosing CBDS (7).

At present, endoscopic retrograde cholangiopancreatography (ERCP) has been widely recognized as the preferred treatment method for CBDS (7, 8). However, ERCP not only leads to complications such as postoperative acute pancreatitis, delayed bleeding, and biliary tract infection, but also in some cases, the stones may not be completely removed. In modern times, with the significant advancement of endoscopic technology, the clearance rate of CBDS should reach over 80%. Combined with mechanical lithotripsy, the success rate can be increased to over 90% (9). That is to say, there are still about 10% of the stones that cannot be removed through ERCP. For CBDS that cannot be removed by ERCP, a biliary stent can be placed first or surgical removal can be performed. If it is possible to determine before the operation which CBDS may not be completely removed by ERCP, it will be of great help in choosing the appropriate treatment method subsequently. Therefore, the aim of this study is to explore the value of MRCP in predicting the success of CBDS removal through ERCP.

## 2 Methods

### 2.1 Ethical considerations

The research was performed according to the Declaration of Helsinki including participants’ consent. The study was approved by the local Ethics Committee.

### 2.2 Patients and study design

Since a small number of CBDS patients may experience failure in ERCP stone removal, if it is possible to predict which patients are highly likely to fail the procedure, it will not only facilitate the clinical doctors in informing the patients and their families in advance for communication, but also help them to discuss the subsequent treatment plans. The preoperative MRCP examination not only enables the diagnosis of CBDS, but also can reveal the size and quantity of the stones, as well as the diameter of the CBD. Therefore, we conducted a multicenter retrospective study on the value of MRCP in assessing the success of ERCP in CBDS patients.

This study included CBDS patients who were hospitalized at First Affiliated Hospital of Wannan Medical College and Affiliated Hospital of Guilin Medical University, and underwent ERCP stone removal treatment from January 2020 to May 2025. The inclusion criteria were as follows: (1) patients who meet the diagnostic criteria for CBDS, (2) preoperative MRCP examination. The exclusion criteria were as follows: (1) ERCP intubation failed and was transferred to surgery, (2) no MRCP examination was conducted at this hospital prior to the surgery, (3) ERCP was performed by doctors with less than 5 years of experience, (4) MRCP did not provide complete information on the size, quantity of the stones, and the widest diameter of the CBD, (5) combined cholangiocarcinoma of the lower segment of the CBD, (6) patients with acute obstructive suppurative cholangitis who only received biliary stents or nasobiliary tubes. All ERCP operators were physicians with many years of experience, aiming to minimize stone extraction failure caused by the operators’ own technical problems. All centers strictly followed the China Clinical Application Management Standards for Endoscopic Diagnosis and Treatment Technologies. During the stone extraction process, the operating physicians could use adjuvant techniques such as balloon dilation, mechanical lithotripsy, and laser lithotripsy according to the actual situation.

All patients who meet the inclusion and exclusion criteria need to record their gender and age. In addition, record the number and diameter of the stones shown by MRCP (for multiple stones, record the largest one), the widest diameter of the CBD, and whether the stone removal was successful during ERCP, as well as the subsequent therapeutic measures taken in case of failure.

**Figure 1:**
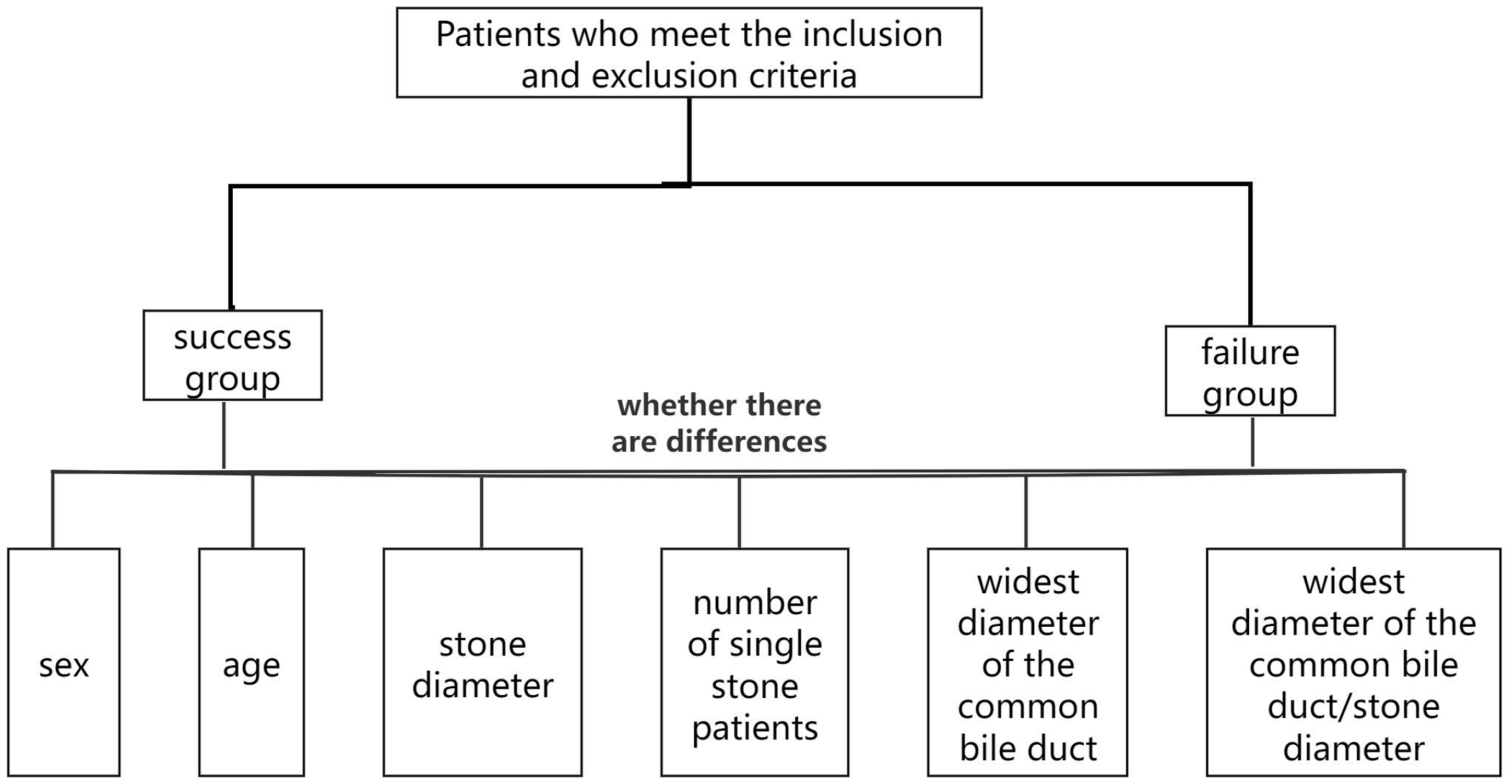
Flowchart.

According to whether ERCP was successful or not, the patients were divided into success group and failure group. Comparing whether there are differences in MRCP-related parameters (including stone diameter, the number of single stone patients, the widest diameter of the CBD, and the widest diameter of the CBD/stone length) between the two groups. Parameters with statistically significant differences were further analyzed using binary multivariate logistic regression (Figure 1).

### 2.3 Statistical analysis

Descriptive data are expressed in terms of median (interquartile range) or counts and percentages. Mann–whitney U test was used for nonparametric tests, and Chi-square test or Fisher’s exact test was used for categorical variables. Binary multivariate logistic regression analysis was used to identify independent risk factors, and ROC curves with area under the curve (AUC) were used to evaluate predictive value. SPSS 22.0 software was used for statistical analysis. A *p*-value < 0.05 indicated statistical significance.

## 3 Results

### 3.1 General information of all CBDS patients

This study included a total of 432 CBDS patients, consisting of 175 males and 257 females. The median (interquartile distance) of age was 68 (57–75) years. The median (interquartile range) of the stone diameter for all patients were 10 (6–16) mm. While the median (interquartile range) of the widest diameter of the CBD for all patients were 14 (12–18) mm. The median (interquartile range) of widest diameter of CBD/stone diameter were 1.40 (1.06–2.00). Among the 432 CBDS patients, the number of patients with a single stone was 240 (55.55%). A total of 395 patients successfully removed all the stones by ERCP, while only 37 patients failed to have their stones removed. The success rate of ERCP stone removal was 91.44%. Among the 37 patients who failed to have stones removed through ERCP, except for one who underwent surgical stone removal, the rest were temporarily fitted with biliary stents and will be reattempted for stone removal after 3 to 6 months (Table 1).

**Table 1:** Clinical features of all CBDS patients.

Variables	Value
Sex (M/F)	175/257
Age (years): M(QR)	68 (57–75)
Stone length: M(QR), mm	10 (6–16)
Number of single stone patients: n(%)	240 (55.55%)
Widest diameter of the CBD: M(QR), mm	14 (12–18)
Widest diameter of the CBD/stone length: M(QR)	1.40 (1.06–2.00)
Number of successful stone removals: n(%)	395 (91.44%)
Treatment measures after failure	
Surgical stone removal: n(%)	1 (2.70%)
Biliary stent implantation: n(%)	36 (97.30%)

M, median; QR, Quartile Range; CBDS, common bile duct stones; CBD, common bile duct.

### 3.2 Comparison of MRCP-related parameters between the success group and failure group

There was no significant difference in the gender (*p* = 0.16) between the two groups. However, the average age of the failure group was generally higher than that of the success group, and the difference was statistically significant (*p* = 0.002). The comparison of MRCP-related parameters between the two groups showed that, except for the number of single gallstone CBDS patients, which did not show statistically significant differences (*p* = 0.174), there were significant differences in the stone diameter (*p* < 0.001), the widest diameter of the CBD (*p* < 0.001), and the widest diameter of the CBD/stone diameter (*p* < 0.001). The stone diameter and the widest diameter of the CBD in the failure group were significantly higher than those in the success group, while the widest diameter of the CBD/stone diameter was significantly lower than that in the success group (Table 2).

**Table 2:** Analysis of the differences between success group and failure group.

Variables	Success group *n* = 395	Failure group *n* = 37	*p*
Sex (M/F)	156/239	19/18	0.160
Age (years): M(QR)	66 (57–75)	73 (68–79)	0.002
Stone diameter: M(QR), mm	9 (6–15)	25 (18–32)	<0.001
Number of single stone patients: n(%)	233 (58.99%)	17 (45.95%)	0.174
Widest diameter of the CBD: M(QR), mm	14 (11–18)	21 (16–25.00)	<0.001
Widest diameter of the CBD/stone diameter: M(QR)	1.45 (1.14–2.00)	0.87 (0.64–1.10)	<0.001

M, median; QR, Quartile Range; CBD, common bile duct.

### 3.3 Binary multivariate logistic regression analysis of independent risk factors for failure of ERCP-guided stone extraction

The parameters in Table 2 that showed significant statistical differences were included in a binary multivariate logistic regression analysis to identify the independent risk factors for predicting ERCP stone extraction failure. Binary multivariate logistic regression analysis revealed that only stone diameter >15 mm was significantly associated with ERCP stone extraction failure (OR = 11.229, 95% CI: 1.576–80.033, *p* = 0.016). Age (OR = 1.027, 95% CI: 0.991–1.066, *p* = 0.145), widest diameter of the CBD>15 mm (OR = 3.254, 95% CI: 0.708–14.951, *p* = 0.129), and widest diameter of the CBD/stone diameter (OR = 0.193, 95% CI: 0.019–1.940, *p* = 0.162) were not identified as independent risk factors. Therefore, stone diameter of CBD > 15 mm is an independent risk factor for ERCP stone extraction failure (Table 3).

**Table 3:** Independent risk factors for ERCP-guided stone extraction failure: binary multivariate logistic regression analysis.

Variables	B	S. E	Wales	df	Sig.	Exp(B)	95%CI
Age	0.027	0.019	2.121	1	0.145	1.027	0.991–1.066
Stone length>15 mm	2.419	1.002	5.826	1	0.016	11.229	1.576–80.033
Widest diameter of the CBD>15 mm	1.080	0.778	2.300	1	0.129	3.254	0.708–14.951
Widest diameter of the CBD/stone length	−1.647	1.178	1.953	1	0.162	0.193	0.019–1.940

CBD, common bile duct.

### 3.4 ROC curve and area under the curve (AUC) for predicting ERCP stone extraction failure based on CBD stone diameter >15 mm

**Figure 2:**
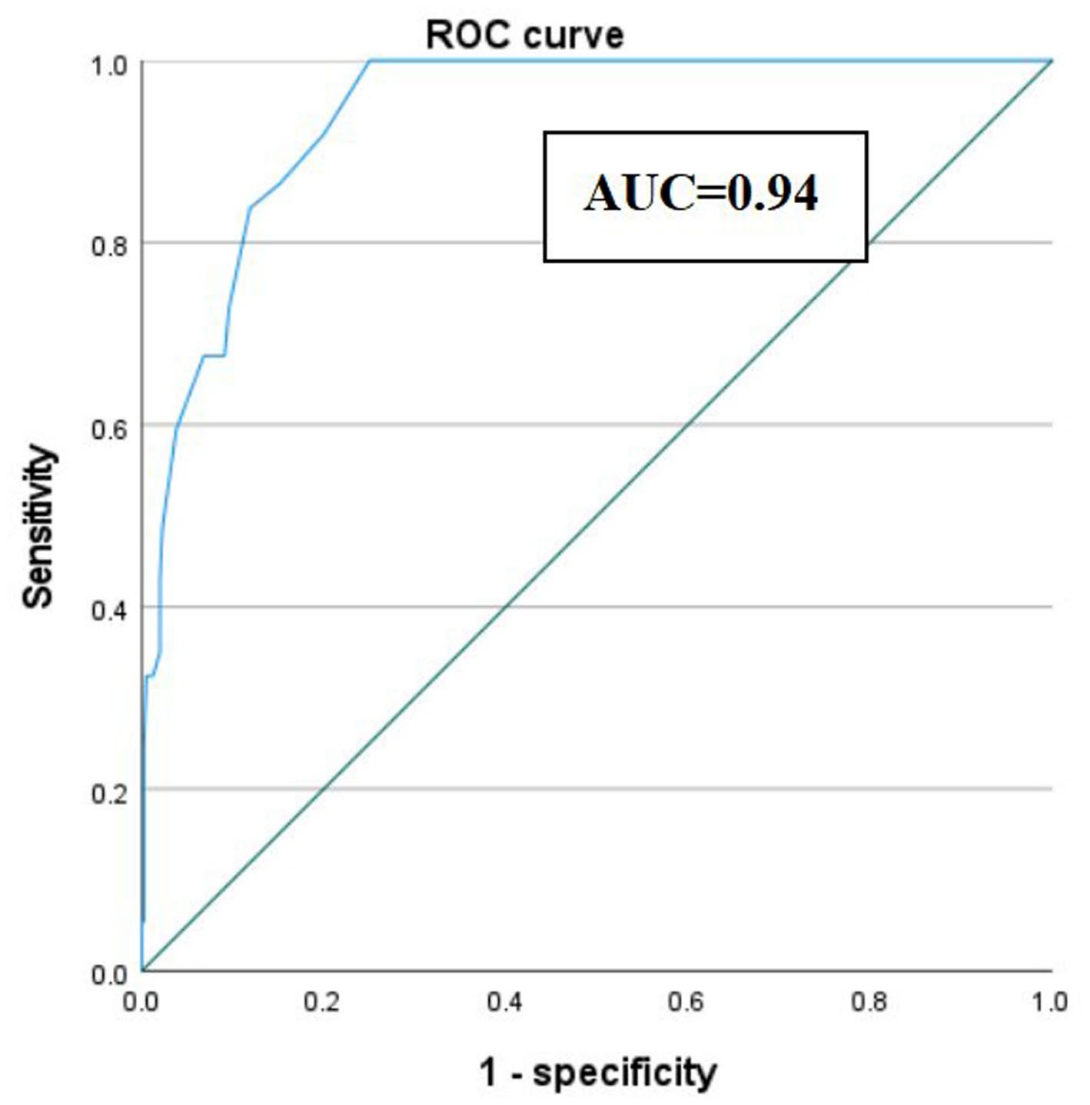
ROC curve and area under the curve (AUC) for predicting failure of endoscopic retrograde cholangiopancreatography (ERCP)-guided stone extraction using the length of common bile duct stone.

We constructed an ROC curve to evaluate the performance of CBD stone diameter >15 mm in predicting ERCP stone extraction failure. The analysis yielded an AUC of 0.94, indicating that this cutoff value has excellent discriminatory ability to identify patients at risk of ERCP failure. When the cutoff value of CBD stone diameter was set at 15 mm, the Youden’s index reached its maximum value of 0.749, corresponding to a sensitivity of 100% and a specificity of 75% (Figure 2).

## 4 Discussion

CBDS is a common type of digestive tract disease. The incidence of CBDS varies by country, ranging from 8 to 18% (3, 10–13). Although most patients with CBDS have no symptoms throughout their lives, 10–25% of them still experience symptoms such as abdominal pain and fever (14, 15). ERCP is currently recognized as the preferred treatment method for CBDS (16, 17). A study published in 2019 showed that the success rate of stone removal through ERCP was 93.18% (18). In this study, the success rate of ERCP stone removal was 91.44%, similar to the aforementioned research. Although the success rate of ERCP in removing CBDS in this study was very high, there were still a few patients who experienced failed stone removal. Accurately analyzing before ERCP which CBDS patients have a high possibility of failing stone removal is beneficial for discussing with the patients and their families in advance about which subsequent treatment plan to choose. MRCP, as the currently recognized and reliable diagnostic method for CBDS, not only can clearly show the size and location of the stones, but also can display the quantity of the stones and the diameter of the CBD.

Previous studies have confirmed that the accuracy rate of MRCP in diagnosing CBDS is over 90% (19–21). This study is focused on the application value of MRCP in predicting whether ERCP can successfully remove all the stones. This study found that there were significant differences between the successful stone removal group and the failure group in terms of the widest width of the CBD, the stone diameter, and the ratio of the two. Further binary multivariate logistic regression analysis showed that a stone diameter >15 mm was an independent risk factor for ERCP stone extraction failure. The sensitivity and specificity of a stone diameter >15 mm for predicting ERCP stone extraction failure were 100 and 75%, respectively. The sensitivity of 100% indicates that all patients who experienced ERCP stone extraction failure (true positive cases) had a CBD stone diameter >15 mm; in other words, if the CBD stone diameter is <15 mm, the probability of successful ERCP stone extraction is extremely high. Some studies have defined stones with a diameter greater than 15 mm as difficult CBDS, indicating a high failure rate of stone removal through ERCP (22, 23). The results of this study are consistent with theirs.

The clinical significance of this study is mainly reflected in two aspects: first, it clarifies that CBD stone diameter >15 mm is the core independent risk factor for ERCP extraction failure, which helps clinicians focus on evaluating stone diameter during preoperative MRCP and formulate individualized treatment plans for high-risk patients. For example, for patients with CBD stone diameter >15 mm, preoperative preparation can be strengthened, such as full evaluation of ampullary function, selection of appropriate endoscopic instruments (such as large-diameter balloon catheters or laser lithotripters), or even consideration of alternative treatment strategies if necessary to improve the success rate. Second, the cutoff value of 15 mm for CBD stone diameter has excellent predictive efficiency, which can be popularized in clinical practice as a simple and easy-to-use predictive standard, especially in primary hospitals where endoscopic technology is relatively limited, helping to improve the level of preoperative risk assessment.

This study has some limitations. Firstly, it is a retrospective study and the number of cases is not large. Secondly, this study excluded some CBDS patients who underwent ERCP but did not have the purpose of stone removal. Furthermore, as this study involved multiple centers, the ERCP operators were not the same person, which might have certain impacts on the results. Therefore, we hope that in the future, more prospective studies involving a larger number of patients can be conducted.

## 5 Conclusion

Preoperative MRCP is of great value in predicting the success of ERCP stone removal. CBD stone diameter >15 mm is a simple, objective, and reliable preoperative predictor for ERCP extraction failure.

## Data Availability

The original contributions presented in the study are included in the article/supplementary material, further inquiries can be directed to the corresponding author.
